# An integrative review of attention biases and their contribution to treatment for anxiety disorders

**DOI:** 10.3389/fpsyg.2015.00968

**Published:** 2015-07-08

**Authors:** Tom J. Barry, Bram Vervliet, Dirk Hermans

**Affiliations:** Centre for the Psychology of Learning and Experimental Psychopathology, Faculty of Psychology and Educational Sciences, University of LeuvenLeuven, Belgium

**Keywords:** anxiety, fear, phobia, attention, exposure, treatment

## Abstract

Models of exposure therapy, one of the key components of cognitive behavioral therapy for anxiety disorders, suggest that attention may play an important role in the extinction of fear and anxiety. Evidence from cognitive research suggests that individual differences may play a causal role in the onset and maintenance of anxiety disorders and so it is also likely to influence treatment. We review the evidence concerning attention and treatment outcomes in anxiety disorders. The evidence reviewed here suggests that that attention biases assessed at pre-treatment might actually predict improved response to treatment, and in particular that prolonged engagement with threat as measured in tasks such as the dot probe is associated with greater reductions in anxious symptoms following treatment. We examine this research within a fear learning framework, considering the possible role of individual differences in attention in the extinction of fear during exposure. Theoretical, experimental and clinical implications are discussed, particularly with reference to the potential for attention bias modification programs in augmenting treatment, and also with reference to how existing research in this area might inform best practice for clinicians.

## Introduction

The clinical utility of cognitive-behavioral therapy (CBT) and in particular exposure therapy, has long been established, especially in terms of its effects on relieving the symptoms associated with anxiety disorders (Hofmann, 2008). Nevertheless, for some clinically anxious people, exposure is insufficient at alleviating their symptoms or, where there is symptom improvement, they may relapse over time (Craske and Mystkowski, 2006). There has been little research into why there are differences between individuals in their responses to treatment although it is perhaps not surprising that such differences exist given the diversity that exists in the biological, psychological, and social pathways to the onset and maintenance of anxiety. Interest is developing in the area of *stratified medicine* and tailoring treatment to individual profiles, rather than assuming that treatment is one-size-fits-all (Lester and Eley, 2013). There is a need, therefore, to better understand the mechanisms that underlie anxiety disorders and how these might influence treatment.

Disorders such as specific phobia, social anxiety disorder (SAD), panic disorder (PD), generalized anxiety disorder (GAD), and post-traumatic stress disorder (PTSD) are characterized by excessively intense, frequent and enduring fear or anxiety concerning the possible occurrence of something terrible. Fear is often an adaptive response to threat, wherein there is a rapid, involuntary, physiological reaction that facilitates the selection and production of an appropriate behavioral or cognitive response (Ekman, 1992). Anxiety is the prospective connection between this fear response and events in the future (Hofmann et al., 2012). Anxious people display hypersensitivity in recognizing, processing and responding to threat-related information even in the absence of actual threat. Where a non-anxious person might rapidly recognize an actual source of threat in their environment, such as an approaching snake, someone with specific phobia for snakes might also display fear and the accompanying neurocognitive response to a shoelace on their kitchen floor. The onset and maintenance of anxiety disorders has been associated with biases in attention, and in particular, a tendency to be easily distracted by potential threats and to maintain attentional focus on these threats at the expense of attending to other, perhaps more important, things in the environment (Bar-Haim et al., 2007).

Cognitive-behavioral therapy for anxiety disorders therefore seeks to reduce fear and anxiety as well as the neurocognitive mechanisms that underlie them (Hofmann, 2008). However, if mechanisms such as attention biases influence the way in which people process and interact with the objects of their fears then we might ask whether they can also influence treatments that rely on interaction with these feared stimuli; and, whether individual differences in attention predict differential responses to treatment. The current review seeks to understand how individual differences in attention might influence treatment.

First we will explain our conceptualisation of attentional biases, whilst also addressing the literature concerning individual differences in attentional biases within anxiety disorders. Second, we will consider the role of attention in the learning that underlies exposure therapy. Then, we will conduct a critical examination of the literature concerning the moderating influence of attentional biases, and any related neurophysiological correlates, on treatment outcomes for anxiety disorders. The results of these studies will be considered within the context of models of the underlying mechanisms involved in exposure (e.g., extinction learning). Finally, the theoretical, experimental, and clinical implications of this examination will be presented. The research in this area remains in its infancy, thus precluding a meta-analysis of the data available, but we hope that by collating and reviewing the present evidence and providing the most up-to-date theoretical perspective, that we might provide inspiration for clinical and research developments.

## Background

### Attentional Biases

Limitations in the capacities of both the visual system and working memory mean that not all information from the environment and from long-term memory can be selected for more detailed processing. Instead, some information is given priority and is selected, and unwanted information is filtered out. From this, appropriate responses to environmental stressors can be produced. Prioritization and selection can be through top–down, voluntary, goal direction or bottom–up, automatic, attentional capture (Weierich et al., 2008). Stimulus processing and attentional selection can also be overt (e.g., through eye movements) or covert (e.g., the orientation of internal attention). For example, a person conducting a top–down overt visual search for a particular flower might covertly filter out the various trees and bushes in a woodland scene. As part of this covert filtering, visual sensitivity might increase for objects more alike one’s expectations of the sought after flower. This would reduce the number of stimuli to be processed during the overt movement of the eyes during the visual search. However, a visually salient, large, red tree in this scene may capture attention in a bottom–up way, irrespective of the viewer’s goals. For anxious people attentional selection appears to be weighted in favor of bottom–up processing – particularly when there is a potentially threatening stimulus in the environment – relative to non-anxious people who can evoke greater top–down attention control (Eysenck et al., 2007). The effect of this is that anxious people have a tendency for their attention to be captured by threat-relevant stimuli over and above other stimuli, even when their attention should be focused elsewhere (McGinnis Deweese et al., 2014). Attentional selection and control is then facilitated by three executive processes: *shifting* between stimuli, thoughts or responses; *updating* attentional selection based on changes in goals; and *inhibiting* attentional engagement with irrelevant stimuli or thoughts (Eysenck et al., 2007).

Research suggests that prior to the onset of and during anxiety disorders, attention can be biased such that there are deficits in *inhibiting* distraction by emotional stimuli, enhanced *shifting* of attention toward or away from these stimuli or deficits in shifting away from threat stimuli once engaged, as well as deficits in *updating* attention based on new information, perhaps pertaining to safety. Biases in attention can therefore be categorized into three components subsumed within two directions (toward or away from threat): engagement with threat, disengagement with threat, and an attentional avoidance of threat (Cisler and Koster, 2010).

#### Engagement with Threat

Anxiety has been associated with facilitated engagement with threat. This means that salient, potentially threatening stimuli can capture the attention of anxious people more readily than non-anxious people, the result of which is that they show rapid movement of their attention toward these stimuli (Eysenck et al., 2007). This is perhaps due to increased utilization of the rapid, subcortical, amygdala-driven “low road” in the processing of emotional stimuli, rather than through use of the higher cortical pathway (LeDoux, 1996). For example, when someone rapidly orientates toward a shoelace on their kitchen floor that they thought was a snake. In this case there is bottom–up attentional capture due to peripheral recognition of the perceptual features of the lace (e.g., its length, color, and shape) and their similarity with the appearance of a snake. This leads to rapid re-orientation of eye-gaze and attentional engagement with this perceived threat.

This bias has been examined in a number of experimental settings. In visual search paradigms anxious people typically show quicker detection of disorder-relevant and threatening targets hidden amongst neutral distractors relative to non-threat targets (Lipp and Waters, 2007; Soares et al., 2009) and relative to the performance of non-anxious people (Rinck et al., 2005). Gaze-tracking research has shown that, relative to non-anxious people, anxious people are more likely to orient their gaze toward threat stimuli before they orient toward neutral stimuli when the two are presented simultaneously (e.g., Calvo and Avero, 2005; Felmingham et al., 2011). There is also some evidence that anxious people orient their gaze toward emotional faces, irrespective of their valence, quicker than do non-anxious people (Holas et al., 2014). In Dot Probe tasks where participants are shown threat and neutral stimulus pairings followed by a geometrical target to which they are to respond, anxious participants have also shown speeded recognition of the target when it follows a threat stimulus, relative to if it follows a neutral stimulus and relative to non-anxious participants (Bar-Haim et al., 2007).

#### Disengagement with Threat

The second attentional bias component concerns disengagement with threat. Research has consistently demonstrated that anxious people show slowed response times or a deficit in disengaging attention from threat-related stimuli, relative to non-anxious people. Anxious participants perform more slowly than controls in visual search experiments where participants must locate neutral targets that are hidden amongst threatening distractors (Gerdes et al., 2008). Anxious people also perform more slowly than controls in Dot Probe trials when the target follows the neutral stimulus rather than the threat stimulus (e.g., Koster et al., 2006) and in spatial cuing trials when a target appears on the opposite side of a computer screen from a preceding threat stimulus (e.g., Cisler and Olatunji, 2010). Persistent engagement with a threat stimulus can be at the expense of attending to other neutral, positive or safety signaling stimuli presented simultaneously, or other stimuli in the environment that require attention. This has been confirmed with eye-tracking measures in both visual search and free-viewing paradigms (e.g., Gerdes et al., 2008; Armstrong et al., 2010) where anxious people have been shown to fixate longer on threat stimuli relative to non-threat stimuli. There has also been some suggestion that in the Stroop task, slowed naming of the color that threat words are presented in is also evidence of difficulties in disengaging attention from threat, however, this has been widely disputed (Williams et al., 1996; Weierich et al., 2008).

The effect of maintained attention toward a threat stimulus is that the person may continue to think or ruminate about the possibility of an approaching threat (Fox et al., 2001) and their attention may then narrow, preventing them from attending to other less salient peripheral cues in their environment (Eysenck et al., 2007). As such they may remain in an anxious state for longer, and they may be prevented from shifting their attention to information that violates their expectancy of an aversive event. For example, in dog phobia, attending to the teeth of a barking dog might prevent one from acknowledging that the dog is attached to a leash and cannot reach them.

#### Attentional Avoidance of Threat

The third component of attentional biases involves avoidance of threat, where the person covertly filters out threat-relevant stimuli or thoughts from attentional selection and overtly avoids attending toward them, should they appear (Hofmann et al., 2012). When an avoidant response is initiated, the scope of attention becomes narrowed which in turn limits the likelihood that peripheral threat stimuli will be recognized (Förster et al., 2006). As such, avoidance prevents the anxious person from encountering and processing information that might violate their expectancy of something aversive happening. To return to the example of the shoelace on the kitchen floor, some anxious individuals, after initially covertly or overtly recognizing the shoelace or snake in their periphery may look away immediately. This prevents them from learning to discriminate snakes from other perceptually similar stimuli, so their generalized fear persists; and if there was a snake, they would never learn that snakes are perhaps not as dangerous as they believe.

Eye-tracking research has shown that if a threat stimulus is presented for a long period of time, anxious people tend to avoid looking at it (Calvo and Avero, 2005). Avoidance is also common when threat images are also associated with feelings of disgust as with pictures of needles in Bloody-Injection-Injury phobia (Armstrong et al., 2013). Implicit within avoidance is that threat is first recognized – although not necessarily overtly or consciously (Ellenbogen and Schwartzman, 2009) – and then later avoided due to top–down redirection (Armstrong and Olatunji, 2012). This has also been demonstrated in conditioning research where, at shorter latencies, participants show orientation toward stimuli that are aversively conditioned (CS+) relative to neutral stimuli (CS-). At longer latencies, however, participants tend to look more toward neutral CS- than toward CS+ (Mulckhuyse et al., 2013).

It is worth stressing that the components of attentional biases should not be considered in isolation or as mutually exclusive from one another (Weierich et al., 2008). Eye-tracking research suggests it is possible to exhibit facilitated engagement with threat immediately after a threat is presented as well as avoidance at later stages of presentation (Armstrong and Olatunji, 2012). It is also possible to distinguish between anxious individuals who have a general tendency to attend toward threat from others who avoid threat (Koster et al., 2006). There may also be people who possess neither of these tendencies but who find it difficult to disengage from threat once it is engaged (Fox et al., 2002). Attentional biases are also not necessary components of anxiety disorders; there are some anxious people who may not exhibit any bias (Berggren and Derakshan, 2013). They are also not specific to anxiety as they may also be present, although qualitatively different, in depressed individuals (Peckham et al., 2010) and persons with eating disorders (Shafran et al., 2007) when disorder-relevant stimuli are presented.

There is overwhelming evidence concerning the presence of attentional biases in anxiety disorders (Bar-Haim et al., 2007; Armstrong and Olatunji, 2012). If attention is seen to play a role in any element of the process of change in treatment, we might expect treatment outcomes to be influenced by these attentional biases. More specifically, given the heterogeneity in attentional biases between anxious people – toward or away from threat; facilitated engagement or deficits in disengagement; any bias or no bias – that the presence of individual differences in attentional biases might produce differential outcomes. A wealth of research exists suggesting that attention can play a crucial role in the learning that is thought to take place during exposure therapy, a principle behavioral component of CBT for anxiety disorders. We will now ask, how does exposure therapy treat anxiety, and what is the role of attention in this process.

### Attention and Exposure Therapy

Exposure therapy is the most efficacious treatment for anxiety disorders (Craske et al., 2008), whether it is administered alone or as one part of a larger CBT program. The learning that occurs during exposure therapy and the reduction in fear and anxiety that is observed is often explained using Pavlovian models of fear conditioning and extinction. In these models, fear develops due to an association between affectively neutral, conditional, stimuli (CS), such as animals, objects, situations and physiological sensations, and aversive, unconditional, stimuli (US), such as anything that might cause physical or psychological harm (e.g., a dog bite, negative judgment, a panic attack). Fear is then expressed when CSs are encountered, the CS–US association is retrieved from memory and subsequent anticipation of the US occurs. Exposure therapy involves repeated exposure to these feared CSs in the absence of the expected aversive US. Exposure is thought to be successful when a person can approach a previously feared stimulus without the concomitant experience of fear.

There are two predominant approaches to explaining the effects of exposure therapy and the mechanism by which fear and anxiety reduce. In habituation or emotional processing models, CS, their aversive meanings and the accompanying emotions, cognitions and behaviors are held as *structures* within memory. The fear evoked by a CS reduces during exposure therapy due to activation and correction of the fear structure as it is updated with new information about the affectively neutral nature of the CS (Foa and Kozak, 1986). This model was later modified to include an inhibitory *safety* structure in order to accommodate research suggesting that fear memories may only be inhibited rather than updated (Foa et al., 2006). Nevertheless, in order to elicit the most fear habituation – and reduce the chances of a return of fear after exposure – the fear structure must be maximally activated during exposure. The presentation of a feared object does not guarantee that functional exposure (awareness and processing of feared stimuli) or extinction of fear will take place (Borkovec and Grayson, 1980). Fully activating the fear structure requires that clients focus their attention entirely on the subject of their exposure and are not given any opportunities for avoidance or distraction and this then facilitates habituation of the fear response.

The alternative approach comes from inhibitory learning models of fear extinction, which suggest that when CSs are encountered in the absence of the US, a new CS–noUS association is formed and this inhibits the original fear eliciting excitatory CS–US association. The original CS–US association remains intact. Under certain conditions it might be retrieved and fear may return and clinical relapse might occur (Craske et al., 2008). In order to fully inhibit the original CS–US fear association, there must be maximal violation of US expectancy. This means that the client learns that the object of their fear is no longer predictive of an aversive consequence. Within this approach, habituation of the fear response is not necessarily indicative of the extent of this new learning. It is essential that this new inhibitory learning is not conditional upon some other element of the exposure. Clients should not attend to any element of the exposure stimulus or its surrounding context that might explain the non-occurrence of the US. For example, whilst being treated for snake phobia, the client must focus on the snake they are being exposed to so that they can learn that snakes are not always dangerous. The client should not, however, distract themselves by not thinking about the snake or by attending to other elements of the clinic room in which they are sat or any features of the snake (e.g., its small size) that might explain why this snake is not dangerous.

#### Attention and Distraction

According to both models of exposure treatment presented here, attending to anything other than the object of an exposure session should lead to worse outcomes relative to engagement with a perceived source of threat, although the models differ in their explanation of why this is the case and the extent to which this can be a problem (e.g., the extent to which this leads to habituation of fear). As such there is a body of research concerning the effects of manipulations of distraction on exposure efficacy. Distraction is typically defined as insufficient attention to the source of a potential threat. It can involve overt redirection of attention toward some threat-irrelevant task, stimulus or non-threatening features of a feared stimulus; or covert redirection of attention and attempts to not think about potential threats (Podinã et al., 2013). There are clear similarities between this definition of distraction and threat-avoidant attention biases.

Research concerning the effects of distracted versus focused attention on exposure outcomes might similarly shed light on the possible effects of attention biases on exposure outcomes. In fact, one study found that participants who were distracted during exposure showed similar fear reductions as participants who were given “natural” exposure with no attention manipulation, relative to a third condition where participants were instructed to focus their attention (Craske et al., 1991). The authors reasoned that anxious people are likely to attend away from and avoid threat during normal or *natural* exposure without a specific therapist-initiated attention manipulation and so they are likely to show similar fear reduction as clients who are distracted by the therapist. Research concerning the effects of distracted versus focused exposure has produced mixed findings. A recent meta-analysis found that overall there was no difference between distracted versus focused exposure in terms of treatment outcome (Podinã et al., 2013). However, the results were in favor of distracted exposure – greater approach of and reduced distress concerning previously feared stimuli following treatment – relative to focused exposure when there were multiple exposure sessions (Oliver and Page, 2008) and when distraction involved client-therapist interaction concerning something irrelevant to the exposure (e.g., Johnstone and Page, 2004). Parrish et al. (2008) suggested that the beneficial effects of distraction may be due to increases in self-efficacy and the belief that threat and the accompanying anxiety could be controlled, however, multiple sessions may be required in order to see this benefit.

Interpreting these data in line with attention biases, we might expect that a tendency to attend away from threat would not necessarily be associated with worse treatment outcome if such avoidance during exposure were accompanied by a belief that threat and/or the fear response can be managed. However, if the immediate post-treatment benefit of attending away from threat is provided by improvements in self-efficacy, rather than enhanced inhibitory learning, there is a possibility that fear might return later if people no longer feel as though they are competent enough to manage threat (Craske et al., 2008). That is not to say that attending toward threat would not be beneficial for the treatment of fear. Waters and Kershaw (2015) recently showed that children with an attention bias toward threat, measured using a Dot Probe task, showed enhanced extinction of fear to a CS, relative to children with an avoidant attention bias. As models of extinction learning and exposure suggest, the reduction of fear requires at least some engagement with the potential sources of threat and subsequent learning about the non-occurrence of anticipated danger.

#### Attention and Context Dependency

The inhibitory model of extinction and exposure was developed following research concerning the fragility of extinction learning and the factors that can lead to a return of fear after extinction. One of the main findings concerned the context dependency of extinction learning and the *renewal* effect. During extinction and exposure, when a feared stimulus is presented and the expected aversive US does not occur, the anxious or fearful person seeks to resolve the conflict between their expectation and reality (Bouton, 2004). One way in which this conflict can be resolved is by attending to details of the context that might explain the non-occurrence of the US. Someone who is snake phobic, for example, might see the hospital room and the presence of clinicians as an explanation for why the snake they are being exposed to is not dangerous. Extinction learning then becomes confined to the context and if there is a change of context after extinction or exposure, fear can return (Rowe and Craske, 1998).

Attending more or less toward the context during exposure sessions should influence the extent of return of fear after exposure. As such we would expect that if people attend away from threat during exposure then they may be encoding more contextual detail rather than focusing on the feared stimulus and the non-occurrence of the US. This would make any extinction learning that develops during exposure to be contextually specific and more susceptible to renewal after treatment. We would expect, therefore, that attention away from threat would be predictive of worse outcomes relative to no bias and attention toward threat, particularly in single session exposure trials when there is little additional opportunity for threat engagement and extinction learning. We would also expect that attention toward threat, and in particular deficits in disengaging from threat, would outperform no bias at all given that there may be attentional narrowing (Eysenck et al., 2007) and reduced contextual encoding in people who attend more toward feared stimuli during exposure sessions.

There has as yet been no direct examination of the effects of attention toward or away from a context on return of fear, however, research using rats found that administration of the drug scopolamine during extinction reduced contextual encoding and return of fear following a context change after extinction (Zelikowsky et al., 2013). Scopolamine, a drug that blocks activity in acetylcholine receptors, is known to reduce attentional capacity, narrow attentional focus and reduce top–down attentional control (Dunne and Hartley, 1986; Klinkenberg et al., 2011). Administration of scopolamine during extinction might reduce peripheral attention and so too the contextual specificity of extinction learning.

#### Attention and Stimulus Generalization

Attention might also influence extinction learning and exposure in terms of stimulus processing and generalization. Besides attending to details in the exposure context, anxious people might also look to some element of the exposure stimulus itself in order to explain why the US has not occurred (Vervliet et al., 2004). In exposure therapy, there is a reliance on perceptually similar stimuli or situations that share only some of the features of the original CS. For example, if someone is bitten by a dog and subsequently develops a dog phobia, then it is very likely that the original dog will not be accessible at the time of treatment. It can be possible to explain the non-occurrence of the US by recognizing the features of the exposure stimulus that were not present in the original CS. For example, if someone is bitten by a black, long-haired, dog such that they develop a fear for dogs, and then they are exposed to a blonde, long-haired dog during treatment, the inhibitory association could be confined to the features in common between these two dogs (e.g., their long hair) and their may be an enduring fear association between the features unique to the original dog that were not present in exposure (e.g., black hair). The more common exposure stimuli are to originally feared or prototypical stimuli, the more robust the extinction learning (Vervliet et al., 2005, 2006).

If someone attends more to the features in common between the original CS and the exposure stimulus, which have previously been associated with threat, then they may not recognize or encode the dissimilarity between these two stimuli and their extinction learning will be more generalizable. We would hypothesize from this that an attention bias toward threat, and in particular deficits in disengagement, would be associated with better outcomes relative to an attention bias away from threat or no bias at all as these biases may influence attention to threat or non-threat stimulus features. However, it might also be possible to initially attend toward the threatening features of a stimulus and to then overtly shift away from and avoid them whilst still covertly engaging with the threat and continuing to think about it. In this case we might expect an attention bias away from threat to outperform no bias at all. This would be similar to interactive distraction conditions where an attention bias away from threat and distraction could both provide an opportunity to manage anxiety levels providing that there is still engagement with threat stimuli or their threatening features.

#### Attention and the Non-Occurrence of the US

It is implicit within models of extinction learning that in exposure people must not only attend to the stimuli or situations to which they are being exposed, but also to the non-occurrence of the aversive event, the US, that they anticipate will occur (Craske et al., 2008). This can be an issue in the case of subtler USs such as in SAD with negative social reactions that are very rarely explicit. In such instances anxious people might attend more to negative thoughts about their performance or their physiology (e.g., concerning their heart rate or their blushing) rather than on the non-occurrence of the imagined negative event. This kind of self-focused attention is thought to underlie disorders such as SAD (Clark and Wells, 1995; Spurr and Stopa, 2002) and PD (Olatunji et al., 2007). Clark and Wells (1995) suggest that self-focused attention prevents people from attending to external information in their environment that might disconfirm their beliefs about the occurrence of negative evaluation after social encounters. We might expect that difficulty in disengaging from threat might be related to maintaining attention internally. This is relative to when someone shows a tendency for facilitated engagement with threat or avoidance of threat where the ability to shift attention is preserved. In these cases, we might expect a shift in attention from internal cues to the non-occurrence of an expected negative outcome.

Attention plays an important role in both the onset and maintenance of anxiety disorders and also in the learning that takes place during their treatment, irrespective of the model one uses to explain the effects of exposure therapy. We will now examine the evidence concerning how attention to threat-relevant stimuli differs between individuals and what the effects of these differences are on the efficacy of exposure therapy.

## Attentional Biases and Treatment Outcomes

Research in this area began indirectly with a number of studies that explored the effects of exposure (alone or as part of a program of CBT) on attention biases. These studies measured attention biases in terms of performance interference on a color naming Stroop task when the words included in the task were disorder-relevant (e.g., spider phobic participants being shown spider, web or crawling). Lavy et al. (1993; *N* = 36) showed evidence of a trend-level correlation between pre-treatment Stroop interference (SI) scores and behavioral avoidance following treatment (*r* = -0.25, *p* = 0.07). We might tentatively interpret this finding as suggesting that greater interference from disorder-relevant words on color-naming in a Stroop task was associated with greater behavioral avoidance following treatment. Put otherwise, a threat-related attention bias was associated with worse treatment outcome. In another study, there was no significant difference in pre-treatment SI scores for social threat words between clients who responded to treatment and those that did not (Mattia et al., 1993; *N* = 33). Participants in this study were given either a pharmaceutical intervention, pill placebo or 12-weeks of group CBT (including sessions of cognitive restructuring, goal setting and exposure). However, the authors did not test whether treatment response was differentially related to SI scores for each of these treatment modalities separately. This makes it impossible to conclude as to the specific relationship between CBT or the exposure component within and SI. van den Hout et al. (1997) also reported no correlation between pre-treatment SI scores and treatment gains in a sample of participants with specific phobia (*N* = 37) who received a single session of exposure treatment. Lundh and Öst (2001) showed that in a sample of social phobia clients who received individual, group or self-administered CBT (*N* = 24) that a greater proportion of treatment responders (78%) showed SI at pre-test relative to non-responders (50%) who “did not show any Stroop Interference before treatment,” although this was not statistically tested. However, the authors did not provide detail on the components of their CBT programs limiting the conclusions that can be made concerning the relationship between attention biases and exposure. In their investigation into the effects of different psychological treatments on SI on motor-vehicle accident related PTSD (*N* = 23), Devineni et al. (2004) showed that pre-treatment SI did not significantly predict the presence or absence of PTSD immediately after treatment or at 3-months follow-up. However, they also report an improvement in PTSD status irrespective of whether participants received CBT or were on a wait-list control. It is meaningless therefore to draw any distinction between treatment responders and non-responders given that it is impossible to differentiate a treatment responder from a control participant whose symptoms abated over time. Also, more recently, researchers have questioned the validity of using SI to measure attentional biases. Performance on the Stroop does not require any overt orientating of attention and it is not possible to determine whether the Stroop is instead drawing upon a covert attentional system. SI may be more indicative of a general threat-processing deficit rather than a deficit in any specific attentional component (Weierich et al., 2008). As such, the aforementioned studies are presented in brief because of their significant methodological limitations and the problematic use of SI to infer attention biases (see Discussion).

In light of these methodological problems, Legerstee et al. (2009) were the first researchers to directly investigate the effects of threat-related attentional biases on treatment outcomes. Children (*N* = 131; age range 8–16 years) who presented with a primary diagnosis of Separation Anxiety Disorder, GAD, SAD, or Specific Phobia were randomized into group or individual (although there was no test of whether either was better than the other) CBT programs. The authors reported that response latencies to a Dot Probe target that followed a severe threat image in severe/neutral image pairings predicted 10–13% of the variance in treatment outcome (*p* = 0.001), even when controlling for pre-treatment anxiety severity. Contrary to their hypotheses, treatment responders were *less* likely to engage their attention toward threat images than non-responders, who were more likely to show difficulties in disengaging their attention from threat. According to the authors, people who tend to inhibit negative information and avoid processing threatening stimuli in detail may respond better to CBT.

Legerstee et al. (2010) replicated this finding in a similar study that separated participants in terms of whether they responded to treatment and how long it took for them to respond. Participants (*N* = 91) had a similar demographic and diagnostic profile to participants in their previous study. Initial responders no longer met diagnostic criteria after 10 sessions of CBT alone and four sessions with their parent; secondary responders were those who continued to meet diagnostic criteria after these initial sessions but who later responded following 10 subsequent sessions of combined child and parent sessions. They found that initial responders showed slower responses on congruent trials where the target followed the threat image in threat/neutral image pairings, than on incongruent trials where the target followed the neutral image. Put otherwise, initial responders showed selective attention away from threat, attending more toward neutral images than to threat images. Secondary responders showed selective attention toward threat with slower responses on incongruent trials than on congruent trials. Treatment non-responders showed no evidence of an attentional bias toward or away from threat before or after treatment. Following treatment, initial and secondary responders showed difficulties disengaging from threat. This confirmed Legerstee et al. (2009) earlier conclusion that exposure might reinforce prolonged engagement with feared stimuli. Participants with a bias in either direction responded better to treatment than people with no bias at all.

Price et al. (2011) investigated whether the direction of attentional selectivity, toward or away from threat, influenced treatment outcome in a sample of adults with SAD (*N* = 24) who underwent eight sessions of CBT including virtual reality exposure. Attentional bias was assessed using a neutral/threat (angry) face Dot Probe task. Mean response times in congruent trials, when the target followed the threat image, were subtracted from incongruent trials, where the target followed the neutral image. Participants with a positive score on this index were classified as being vigilant toward threat whereas a negative score meant they were avoidant of threat. Avoidant attention biases at pre-treatment were associated with worse post-treatment symptom scores relative to vigilant threat biases. A tendency to attend more toward threat was associated with improved outcomes relative to attending away from threat. The authors reasoned that avoidance of threat was analogous to distraction and poor engagement with exposure and worsened extinction learning. Further analysis suggested that the effect of an avoidant attention bias on treatment outcome was independent of the severity of this bias. However, for those with a vigilant threat bias, higher scores were associated with worse response to treatment. These findings suggest that even though generally attending toward threat may be beneficial, there is also variability within this bias, such that being highly vigilant and perhaps not being able to attend anywhere other than toward threat can be detrimental.

The findings of Price et al. (2011) were replicated in a sample of children with a principle diagnosis of GAD or SAD (*N* = 35) that received a group CBT program (Waters et al., 2012). After 10 weeks of treatment, participants with an attention bias toward threat, assessed using the angry-face Dot Probe task, showed lower parent-rated symptom severity relative to children with an attention bias away from threat. Children with an attention bias toward threat were also less likely to meet diagnostic criteria at post-treatment. Waters et al. (2012) added that the relationship between attention biases and treatment response appeared to be independent of the severity of pre-treatment disorder severity.

Finally, Niles et al. (2013) gave participants (*N* = 22) a 12-session course of CBT or ACT for social phobia. They used a Spatial Cuing task to measure attentional biases. In their variant of the Spatial Cuing task, threatening (angry or disapproving) faces or images of neutral household objects were presented in the center of the screen and this was then followed by a target letter above, below, or to the left or right. The time participants took to identify the target letter was then averaged across both threat categories. Participants who showed slower reaction times identifying targets that were preceded by a threatening face (interpreted as difficulty disengaging from threat) also showed greater improvement in clinician-rated fear and avoidance scores after treatment, relative to participants who showed no response slowing. There was also a trend toward the same relationship with self-report symptoms too. Attention toward threat, and in particular difficulty disengaging with threat, was associated with improved response to treatment relative to if a client showed no threat-related attention bias.

### Attention-Like Traits and Treatment Outcome

People who selectively attend toward threat may also be those who adopt a *monitoring* coping style in managing their anxiety and who seek knowledge about sources of threat and make greater attempts at processing threat stimuli (Mobini and Grant, 2007). Alternatively, people who selectively attend away from threat – or who adopt a *blunting* coping style – may be more likely to inhibit threatening information and avoid fully engaging with exposure stimuli or situations (Mobini and Grant, 2007). There have been a number of studies concerned with exploring whether monitoring or blunting coping styles, measured using self-report questionnaires, were related to treatment outcomes (Steketee et al., 1989; Muris et al., 1993a,b, 1995; Antony et al., 2001). However, the findings in this area have been mixed. Steketee et al. (1989) found that monitors, relative to blunters, had greater habituation in heart rate, within and between exposure sessions. However, Muris et al. (1993a,b, 1995) showed that blunters, relative to monitors, improved most in self-report symptom measures and behavioral approach following a one-session exposure treatment. These studies do not include a crucial control group of people who were neither monitors nor blunters, but these data would suggest that monitoring and blunting, or at least self-reporting as such, are both associated with improved treatment outcomes at least immediately post-treatment.

### The Neurophysiology of Attention and Treatment Outcomes

#### Neurological Correlates of Treatment Response

A number of studies have explored the neural correlates of exposure outcomes and in particular how areas of the brain associated with attentional engagement might relate to treatment outcome. Improved treatment outcome has been associated with increased activity in the hippocampus at pre-treatment baseline tests when participants are processing CS–US relationships and in particular when appraising the CS as threatening and predictive of the US (Lueken et al., 2013). Increased volume and activation of the hippocampus has also been associated with an attention bias toward threat faces in a Dot Probe task (Fani et al., 2013). More specifically, deficits in disengagement from threat have been associated with sustained activity in the hippocampus even after attention has been directed away from threat and toward a neutral stimulus (Price et al., 2014). Research suggests that the hippocampus is part of a network of brain regions, including the ventral medial prefrontal cortex and the amygdala, that are responsible for fear acquisition and extinction. In particular, the hippocampus seems to be involved in discriminating new information from information that has previously been learnt (Milad and Quirk, 2012). Increased hippocampal activity and maintenance of attention toward a threat may be associated with increased efforts to retrieve previous learning concerning the CS–US contingency and integrate this with current information about the CS. This new learning in the hippocampus could then be fed into other regions of the network in provoking fear excitation or inhibition depending on whether or not the potential threat that was attended to resulted in the aversive consequence that was anticipated.

Research from pharmaceutical augmentation studies can also shed further light on the possible role of attentional biases in treatment where the pharmaceuticals in question are known to influence the neural attention system. The eye region of the face represents a particularly salient signal for threat for people with SAD. Overt gaze fixation to this region of the face is associated with heightened physiological and amygdala activity (e.g., Schneier et al., 2009; Wieser et al., 2009). The amygdala is thought to play an important role in the expression of fear such that heightened activity in this region has been associated with the anticipation and experience of an aversive event (Milad and Quirk, 2012). Intranasal administration of oxytocin has been associated with increased frequency and duration of fixations on the eye region of the face during free-viewing in a gaze-tracking procedure (Guastella et al., 2008). This might be because oxytocin can reduce activity in the amygdala in response to threat-related faces (Labuschagne et al., 2010). Oxytocin administration has also been associated with enhanced response to exposure therapy in SAD with greater improvement on self-report symptom scores (Guastella et al., 2009). Under the influence of oxytocin, amygdala activity and so too the physiological fear response may be reduced and so socially anxious people are better able to spend more time attending to the eye region of the face. This might in turn facilitate violation of expectancy of negative social judgment and then also lead to clinical improvement.

#### Genetic Correlates of Treatment Response

Lester and Eley (2013) present a review of genetic moderators of treatment response for anxiety disorders. Within this review, there are several particularly relevant examples. First, the short allele, low-expression, polymorphism in the gene that codes for the protein that transports serotonin from one neuron to another (5-HTTLPR) has been associated with improved response to treatment (e.g., Eley et al., 2012). This particular polymorphism is associated with increased serotonin in the cleft between neurons and this has been associated with increased reactivity to stress and increased attention bias for emotional stimuli (Pergamin-Hight et al., 2012). In particular, the low expression polymorphism and greater availability of serotonin in the synaptic cleft has been associated with increased attention toward threat in an angry-face Dot Probe task (Pérez-Edgar et al., 2010; Lonsdorf et al., 2014). The low-expression polymorphism has also been associated with a stronger attention bias toward threat after an attention bias modification treatment (ABMT) procedure designed to train attention toward threat, relative to the high-expression form (Fox et al., 2011). People with the low-expression form may be more likely to show an attention bias toward threat and therefore also improved response treatment.

## Summary of Findings

There is some conflict in the literature concerning attention biases and treatment outcome (see **Table 1** for a summary). There are a number of studies which illustrate how a tendency to selectively attend toward threat is predictive of improved response to exposure treatment relative to attending away from threat (Price et al., 2011; Waters et al., 2012) and relative to not having any particular bias (Lundh and Öst, 2001; Legerstee et al., 2010; Niles et al., 2013). These data receive additional support from neurological and genetic research that suggest that patterns of neural activation and chemical transmission associated with enhanced attentional engagement and deficits in attentional disengagement from threat are associated with greater benefit from treatment (Guastella et al., 2009; Eley et al., 2012; Lueken et al., 2013). Conversely, there is also some evidence to suggest that a tendency to avoid threat is associated with improved response to treatment relative to attending toward threat (Legerstee et al., 2009) or having no bias in either direction (Legerstee et al., 2010). These data gain partial support from self-report data from the domain of coping styles wherein people who report a tendency to blunt emotional information and avoid attending to threat show better responding to treatment relative to people who monitor and engage with threat (Muris et al., 1993a,b, 1995; Antony et al., 2001).

**Table 1:** A summary of research exploring the relationship between pre-treatment attentional bias and treatment outcomes following CBT for a range of anxiety disorders.

	Sample	Measures		Treatment		Main findings
		Attention	Outcomes	Conditions	No. sessions	
Lavy et al. (1993)	Adults; SP (Spiders); *N* = 36	Stroop	Behavioral approach test (BAT)	Exposure with or without elaboration	1	Worse outcomes for people with SI
Mattia et al. (1993)	Adults; SAD; *N* = 33	Stroop	Clinician severity rating (CSR)	Drug vs. CBT vs. placebo	12	No significant effects
van den Hout et al. (1997)	Adults; SP (Spiders); *N* = 37	Stroop	Symptom scale (SPQ); BAT	Exposure alone	1	No significant effects
Lundh and Öst (2001)	Adults; SAD; *N* = 24	Stroop	Diagnostic interview (ADIS)	Individual, group or self-administered CBT	12 or 3 months with manual	Treatment responders more likely to show SI
Devineni et al. (2004)	Adults; PTSD; *N* = 23	Stroop	Symptom scale (CAPS)	CBT vs. group support vs. wait-list	8–12	No significant effects
Legerstee et al. (2009)	Children; mixed; *N* = 131	Dot Probe	Diagnostic interview (ADIS-C)	Individual (69%) or group (31%) CBT	Not given	Treatment responders attend *away* from threat
Legerstee et al. (2010)	Children; mixed *N* = 91	Dot Probe	Diagnostic interview (ADIS-C)	Phase 1: individual CBT Phase 2: parent–child CBT for phase 1 non-responders	10 in phase 1; 4 in phase 2	Initial responders attend *away*; secondary responders attend *toward*; non-responders, no bias
Price et al. (2011)	Adults; SAD; *N* = 24	Dot Probe	Self-report symptom scale (LSAS)	CBT with virtual exposure	8	Symptom reduction associated with attention *toward* threat
Waters et al. (2012)	Children; GAD/SAD; *N* = 35	Dot Probe	Self-report symptom scale (SCAS-P); ADIS-C	Group CBT	10	Symptom reduction associated with attention *toward* threat
Niles et al. (2013)	Adults; SAD; *N* = 22	Spatial cuing	Clinician rated fear and avoidance; LSAS, SIAS, and SPS composite	CBT vs. ACT	12	Symptom reduction associated with attention *toward* threat (*deficit in disengagement*)


*SAD, social anxiety disorder; PTSD, post-traumatic stress disorder; GAD, generalized anxiety disorder; CBT, cognitive-behavioral therapy; ACT, acceptance and commitment therapy; SPQ, spider phobia questionnaire; ADIS (-C), Anxiety Disorder Interview Schedule (-Child version); CAPS, Clinician Administered PTSD Scale; LSAS, Liebowitz Social Anxiety Scale; SCAS-P, Spence Children’s Anxiety Scale – Parent Version; SIAS, Social Interaction Anxiety Scale; SPS, Social Phobia Scale.*

Several earlier studies, prior to the development of more robust measures of attentional selectivity such as the Dot Probe task or the Spatial Cuing task, showed that interference in the Stroop task – and presumably therefore also attention toward threat – was either not associated with treatment outcome (Mattia et al., 1993; van den Hout et al., 1997; Devineni et al., 2004) or was associated with worsened outcome relative to people who showed no interference/bias (Lavy et al., 1993).

Based on the most recent data, the majority of the literature using more valid measures of attentional biases such as the Dot Probe and Spatial Cuing task, seems to suggest that an attention bias toward or away from threat is associated with improved outcome relative to no bias at all. Furthermore, there is some evidence that even when these two biases lead to the same treatment response, they may differ in terms of how rapidly they lead to this response (Legerstee et al., 2010).

## Discussion

Cognitive-behavioral therapy for anxiety disorders is imperfect; relapse can occur. We present one possible cognitive variable in attentional biases that might explain individual differences in the learning that takes place during a crucial component of CBT for anxiety disorder, exposure to feared stimuli, and in so doing might also explain differences in treatment response. The evidence presented in this review suggests that a tendency to preferentially attend toward or away from threat, relative to an equal distribution of attention irrespective of threat, results in greater reduction in clinical symptoms and diagnoses following CBT for children and adults with a range of anxiety disorders. The idea that a neurocognitive factor such as an attention bias that is also associated with the development and maintenance of disorder, is similar to the findings of a recent review of genetic predictors of treatment response wherein the same genes that are associated with greater risk for anxiety are also associated with the greatest improvement following psychotherapy (Lester and Eley, 2013). One of the reasons that attention might influence treatment outcome is through the role that attention plays in the exposure component of CBT. It is possible, although not directly tested in any of the reviewed studies, that a threat-related attention bias alters processing of feared stimuli, the contexts that surround them and their relationships with expected aversive events. In exposure it is essential that clients learn that the things they are afraid of are not necessarily as bad as they expect rather than to learn that they might not be bad because they are in a safe environment or because the thing they are encountering is not the same as the thing to which they originally acquired fear or to other similar things they might encounter after treatment. As such, biases in attentional selection might facilitate greater engagement with threat and increase the possibility that a person might learn that feared stimuli or their fear-evoking features are not necessarily predictive of something aversive occurring. Engagement with threat stimuli and their threat-associated features might also narrow attention and reduce engagement with safe or non-threatening elements of a context or a stimulus’ features that might predict the non-occurrence of the US. Interestingly, this does not appear to be universally beneficial, as Price et al. (2011) reported that though attention toward threat appears to be beneficial, a particularly severe bias toward threat was associated with worse outcomes.

It is important to reconcile the apparent conflict between data that suggest that both an attention bias toward threat and away from threat can both be beneficial to a client. There is evidence from Dot Probe and eye-tracking studies that even where an attention bias away from threat might appear to be present, that this bias is actually characterized by rapid orientation or engagement with threat and then subsequent disengagement and avoidance of threat (Koster et al., 2006; Armstrong and Olatunji, 2012). In a Dot Probe task with 500 ms cue presentations, as is the case in all of the reviewed studies that use that measure, participants are able to rapidly engage with the threat stimulus and then rapidly shift their attention to the opposite neutral stimulus and so respond to any target that follows this neutral stimulus as though they had avoided the threat entirely (Hallion and Ruscio, 2011). A fMRI study showed that the neurological correlates of engagement with threat and of CS–US appraisal continue to show sustained activity even when attention is no longer overtly directed toward threat (Price et al., 2014). Perhaps even when there is overt orientation of the eyes away from threat, covert attention toward threat can persist. In this respect the distinction between persons who appear to have a bias toward or away from threat may not be as clear-cut as reaction time based measures often suggest. More advanced methodology such as eye-tracking or neuroimaging are needed to more accurately examine how these patterns of attention are related to exposure learning.

Another way to reconcile the apparent conflict between attention biases toward and away from threat both being beneficial to treatment relates to the literature concerning focused versus distracted exposure. It may be the case that attending away from threat allows people to continue to engage with the exposure session whilst preventing them from being overwhelmed with fear, in the same way that distracted exposure can enhance treatment benefit (Parrish et al., 2008). Being overwhelmed could prevent anxious people from engaging fully with processing feared stimuli or even prevent them from learning anything.

There is also some variability between the reviewed studies concerning whether they show any effect of attention on treatment outcome. Some of the earlier studies using the emotional Stroop task to measure threat interference showed no association between supposed attentional biases and treatment outcome. However, more recent interpretations of Stroop interference are that it reflects a general deficit in processing of threat rather than any component of attention biases (Weierich et al., 2008). Furthermore, the absence of Stroop interference does not necessarily disqualify someone from having an attention bias as it may also be the case that they exhibit a threat-avoidant attention bias and enhanced disengagement from threat relative to more vigilant people. Also, in some of these studies participants were aggregated across treatment conditions into overall treatment response groups (e.g., Mattia et al., 1993; Lundh and Öst, 2001). As such, caution should be applied to any research where Stroop interference is used to measure threat-related attention biases. More sensitive measures of overt attention such as the Dot Probe and Spatial Cuing tasks revealed significant associations between attention and treatment outcome.

Some of the data presented herein suggests that having no predominant bias toward or away from threat is predictive of worse treatment outcomes relative to the presence of attentional biases in either direction. Despite a consensus within the attention bias modification literature that attention biases must be reduced in the same way that other symptoms are (Hakamata et al., 2010; Hallion and Ruscio, 2011; Beard et al., 2012), the research presented here suggests that attentional biases can actually facilitate clinical improvement. It may even be the case that threat-related attention biases are a necessary component of CBT for anxiety disorders given that some studies showed that having no bias at all leads to no response to treatment.

If it is possible to improve treatment outcome by attending more or less toward threat then it may be the case that procedures that modify attentional biases could be used to augment clinical improvement through exposure. Several studies have been conducted to investigate the possible augmenting effects of ABMT on CBT. Studies have shown that training attention away from threat (e.g., higher proportion of Dot Probe cues following neutral stimuli) can improve treatment response relative to CBT alone (Schechner et al., 2014) and CBT with a placebo ABMT (Riemann et al., 2013; Kuckertz et al., 2014). Amir and Taylor (2012) found that reductions in the bias toward threat from pre- to post- treatment, following ABMT training attention away from threat, was predictive of symptom improvement. Kuckertz et al. (2014) showed that a threat-avoidant bias at pre-treatment led to greatest clinical improvement after their ABMT/CBT augmentative program relative to people with a bias toward threat. However, Rapee et al. (2013) found no effect of their ABMT on clinical outcomes, although they also observed no change in the bias from pre to post-treatment. Two studies combining training attention toward positive stimuli (e.g., higher proportion of Dot Probe cues following happy faces) with one session exposure (Waters et al., 2014) or a larger CBT program (Britton et al., 2013) found no significant effects on clinical outcomes beyond CBT alone.

However, in these studies the ABMT is administered concurrently with each CBT session making it impossible to conclude as to the effects of ABMT and changes in attention on subsequent learning during treatment. Also, the authors did not include an additional comparison group wherein attention was trained toward threat, although it was not within the purpose of their studies to include such a control. This would allow us to conclude as to whether attentional training in either direction leads to better treatment response relative to placebo-ABMT or no ABMT, or whether a bias in a particular direction leads to better response. Nevertheless, there is some promising evidence that augmenting CBT with ABMT might lead to improved outcomes and in particular that training people to attend away from threat might be most beneficial. Future research must now explore how changes in attention biases, as a result of ABMT, might influence the way people learn during other aspects of CBT such as during exposures.

An interesting adjunct to this is that some of the studies reviewed here suggest that not all anxious people possess a threat-related attention bias and if they do it may not be in the same direction. Unaccounted for variability in the direction and extent of pre-existing individual differences in attentional biases in participants of ABMT trials might explain why ABMT shows such small effect sizes as it may be more difficult to train people’s attention in the opposite direction to their existing biases and where there is already an attention bias away from threat it might be more difficult to influence anxiety symptoms by reinforcing this bias (Hallion and Ruscio, 2011). It is most often the case that in ABMT trials participants are distributed into treatment groups irrespective of whether any attention bias exists or not and irrespective of the direction of that bias should it exist (e.g., Amir et al., 2008; Schmidt et al., 2009). The data reviewed here suggests that ABMT research should give greater consideration to pre-existing biases prior to treatment as well as the interacting role that attention biases might play in other elements of CBT. ABMT in conjunction with CBT may not be necessary for people with an attention bias toward threat as they may show a good response to treatment without it. ABMT offered prior to CBT might help train people with no bias to engage more toward threat.

Although it is beyond the scope of the present review to consider how similar processes might operate within other treatments for anxiety, it is worth mentioning that similar processes might operate in treatments such as acceptance and commitment therapy (ACT). In ACT exposure to the sources of one’s fears and reductions in avoidance of fear-evoking stimuli are crucial. Niles et al. (2013) found that pre-treatment attention biases were a better predictor of treatment outcome for participants who underwent CBT than for participants who underwent ACT. They hypothesize that ACT may specifically target and modify attentional processes and so the effects of individuals differences on treatment outcome are reduced. This suggests that individual differences in attention may only influence outcomes in CBT because there is insufficient modification of attention in existing CBT packages. ACT-based research also suggests that high levels of trait avoidance, similar to the attention-like trait of blunting, is predictive of lower engagement with the exposure components of treatment (Levitt et al., 2004). Future research could examine the benefit to CBT of incorporating some element of the attentional training that exists in approaches such as ACT.

### Clinical Implications

The evidence reviewed here has several implications for clinical practice. We present one example of how individual difference variables can influence treatment outcomes. This has rather broad implications for clinical practice in suggesting that greater care should be taken in tailoring treatment to individual neurocognitive profiles rather than to try and fit everyone into the same treatment. More specifically, similar to Parrish et al. (2008) who showed in their review of the effects of distraction on exposure outcome, we have shown that there is a range of evidence suggesting that threat-related biases in attention are not necessarily as negative as has been suggested elsewhere and there should be less clinical emphasis placed on resolving these biases where they exist. As was suggested by Legerstee et al. (2010) it may even be necessary to train attentional biases toward or away from threat in order to produce the best response to treatment. Nevertheless, clinicians may find it beneficial to measure attention biases prior to treatment in order to gear treatment to individual cognitive profiles. In the presence of no attention bias or an attention bias away from threat, it might be beneficial for clinicians to ensure that clients are focused on the exposure stimuli and not the context of exposure, as well as the threatening features of exposure stimuli and its commonalities with other similar stimuli. In the case of attention toward threat it would be important to ensure that clients aren’t overwhelmed by their anxiety, and in particular cases of SAD and PD, it would be important to make sure that clients do not focus too much on internal symptoms but also acknowledge that the anticipated negative outcome is not occurring. However, some important issues must be examined further before more meaningful conclusions and recommendations can be given.

### Future Research

One important omission in the existing literature concerns whether any of the observed effects of attention on treatment outcome are preserved at follow-up assessments. This is particularly important because it has often been shown that people who respond best to treatment also often continue to show selective attention toward threat after treatment (Legerstee et al., 2009, 2010). Existing data concerning the potential causative role of attentional biases in the development and maintenance of anxiety (Bar-Haim et al., 2007; Van Bockstaele et al., 2014) might suggest that those people who continue to show a bias at post-treatment would also be the most likely to relapse. For example, threat-related attentional biases have been shown to be predictive of increased reactivity to social stressors (Fox et al., 2010) and stimuli related to specific phobias (Van Bockstaele et al., 2011). Prospective research has also shown that children who show an attentional bias toward threat are more likely to develop social withdrawal typical of SAD in adolescence (Pérez-Edgar et al., 2011). If attention biases develop or are reinforced by exposure treatment, this could be a mechanism by which relapse occurs, even if treatment is seen to be effective immediately after treatment completion.

However, preferentially attending to previously feared stimuli instead of neutral stimuli may not be maladaptive providing that once a stimulus is attended to, there is not an overestimation that something negative might subsequently occur. Unless increased attention toward threat was also associated with anticipation of an aversive event, then we might not expect any return of fear to occur. As Legerstee et al. (2009) suggest, exposure therapy might encourage people to attend toward supposedly threatening stimuli but that is not to say that it encourages people to anticipate threat after encountering a once feared stimulus. It may also be the case that the attentional system operates similarly to the valence system in so far as it is possible to show extinction of fear and new learning about the contingency between a CS and an aversive US but that is not to say that once feared stimuli will show any change in pleasantness after extinction or exposure (Hermans et al., 2002; Dirikx et al., 2004) just as people may still show some preferential attentional tendency around them.

The research to date also lacks an analysis of any possible interactive effects of attentional biases with diagnosis on treatment outcome. Most studies include a broad range of anxiety disorders and lack the sample size to investigate whether the effects of attentional biases on treatment outcome are specific to a given anxiety diagnosis or whether the effects are transdiagnostic. There is evidence to suggest that there is some specificity in the direction and latency of attentional biases between anxiety disorders (Armstrong and Olatunji, 2012) and so we might also expect differential effects on treatment. Additionally, most of the studies reviewed here include exposure within an overarching CBT program. It is important that further research clarifies whether the effects of attentional biases on treatment outcome are specific to exposure and extinction learning or are due to an interaction between attention and some other element of CBT. What is also lacking in many of the studies reviewed here is a sample of participants who exhibited no attention bias in any particular direction. This third group can act as a vital comparison in determining whether any bias is better than no bias in terms of positively influencing treatment outcome. Future research could also assess the potential relationship between trait-based individual differences in the scope of attention (narrow or broad), perhaps using the Navon task (see Navon, 1977), and the direction of attention biases in predicting treatment outcome. It may be the case that among those who preferentially attend toward threat, only those who also show a tendency for narrowed attentional focus would show improved response to treatment relative to those with a broader focus of attention. This is because the broad group may be more likely to also process contextual cues that might explain the non-occurrence of the US, whereas the narrow group may only attend to the threat stimulus. Where possible Dot Probe and Spatial Cuing tasks should also use disorder-congruent and perhaps also individually selected stimuli so as to provide the most accurate picture of the extent of individual attention biases (Pergamin-Hight et al., 2015). Much of this work can also be achieved experimentally, similarly to the study performed by Waters and Kershaw (2015) where fear is installed and extinguished in a laboratory setting, and the effects of individual differences in attention are explored.

Nevertheless, research must now directly examine, through experimental and clinical investigation, *why* individual differences in attentional biases can influence treatment outcome. There must be a move away from reaction time measures to more robust measurement techniques that allow for direct, online, measurement of overt and covert attention before and during exposures. Eye-tracking methodology could provide such a measure of overt attention and neuroimaging might be used to assess covert attention. Pre-treatment fixation data could be used to predict subsequent treatment response in the same way that Dot Probe data has to-date. This could be coupled with neuroimaging data to assess whether the relationship between overt attention toward or away from threat and treatment outcome is in turn related to neural patterns of covert threat engagement. We might also measure fixation and neural activity *during* exposures. From this it would be possible to relate pre-treatment attention biases to actual visual behavior and covert engagement during exposure and to relate these variables to fear within- and between- sessions as well as overall clinical response at post-treatment and follow-up. This would make it possible to test whether people with an attention bias show better treatment response than people with no attention bias because they spend more time processing exposure stimuli rather than to distractors in the environment, contexts or safe stimulus features.

## Conclusion

In order to further improve already efficacious treatments, it is important that there is now a move within clinical psychology from a broad perspective where treatment modalities compete against one another for superiority, to one in which we consider which treatments work best for whom and why. It is important that we now consider how the individual differences that underlie disorder interact with existing treatments in order to better understand the mechanisms by which these treatments work. This knowledge can be used to not only guide the prescription of treatment but also to enhance some element of treatment. More specifically, it is also important that there is a move away from considering biases in attention – and no doubt cognitive biases in general – as universally negative, and a move toward considering them from a functional perspective where they can be positive in certain situations where enhanced threat processing and reduced peripheral processing is required.

## Conflict of Interest Statement

The authors declare that the research was conducted in the absence of any commercial or financial relationships that could be construed as a potential conflict of interest.
